# Protective Effects of Peroxiredoxin 4 (PRDX4) on Cholestatic Liver Injury

**DOI:** 10.3390/ijms19092509

**Published:** 2018-08-24

**Authors:** Jing Zhang, Xin Guo, Taiji Hamada, Seiya Yokoyama, Yuka Nakamura, Jianbo Zheng, Nozomu Kurose, Yasuhito Ishigaki, Hidetaka Uramoto, Akihide Tanimoto, Sohsuke Yamada

**Affiliations:** 1 Department of Pathology and Laboratory Medicine, Kanazawa Medical University, 1-1 Uchinada, Ishikawa 920-0293, Japan; jing2016@m.kufm.kagoshima-u.ac.jp (J.Z.); zjb306919303@gmail.com (J.Z.); k-nozomu@kanazawa-med.ac.jp (N.K.); 2 Department of Pathology, Kagoshima University Graduate School of Medical and Dental Sciences, Kagoshima 890-8544, Japan; tham4506@m3.kufm.kagoshima-u.ac.jp (T.H.); yokoyama@m3.kufm.kagoshima-u.ac.jp (S.Y.); akit09@m3.kufm.kagoshima-u.ac.jp (A.T.); 3 Medical Research Institute, Kanazawa Medical University, 1-1 Uchinada, Ishikawa 920-0293, Japan; yuka-n@kanazawa-med.ac.jp (Y.N.); ishigaki@kanazawa-med.ac.jp (Y.I.); 4 Department of Thoracic Surgery, Kanazawa Medical University, 1-1 Uchinada, Ishikawa 920-0293, Japan; hidetaka@kanazawa-med.ac.jp

**Keywords:** PRDX4, liver injury, oxidative stress, cholestasis, BDL

## Abstract

Accumulating evidence indicates that oxidative stress plays a critical role in initiating the progression of inflammatory and fibrotic liver diseases, including cholestatic hepatitis. Peroxiredoxin 4 (PRDX4) is a secretory antioxidase that protects against oxidative damage by scavenging reactive oxygen species (ROS) in both the intracellular compartments and extracellular space. In this study, we examined the in vivo net effects of PRDX4 overexpression in a murine model of cholestasis. To induce cholestatic liver injury, we subjected C57BL/6J wild-type (WT) or human PRDX4 (hPRDX4) transgenic (Tg) mice to sham or bile duct ligation (BDL) surgery for seven days. Our results showed that the liver necrosis area was significantly suppressed in Tg BDL mice with a reduction in the severity of liver injuries. Furthermore, PRDX4 overexpression markedly reduced local and systemic oxidative stress generated by BDL. In addition, suppression of inflammatory cell infiltration, reduced proliferation of hepatocytes and intrahepatic bile ducts, and less fibrosis were also found in the liver of Tg BDL mice, along with a reduced mortality rate after BDL surgery. Interestingly, the composition of the hepatic bile acids (BAs) was more beneficial for Tg BDL mice than for WT BDL mice, suggesting that PRDX4 overexpression may affect BA metabolism during cholestasis. These features indicate that PRDX4 plays an important role in protecting against liver injury following BDL and might be a promising therapeutic modality for cholestatic diseases.

## 1. Introduction

Cholestasis is a chronic, progressive cholangiopathies with a broad etiology, characterized by hepatocellular injury, bile duct proliferation, and fibrosis, ultimately resulting in liver failure [1]. Although ursodeoxycholic acid and/or anti-inflammatory drug therapies show positive outcomes for some types of cholestasis, most patients with cholestasis must resort to a liver transplant [2,3]. A further understanding of the pathological mechanism underlying cholestasis may encourage the development of novel therapy strategies that control the progression of the disease and help improve its prognosis.

Given that oxidative stress caused by extremely high concentrations of bile acids (BAs) and the infiltration of a large number of inflammatory cells have been detected during cholestasis, many studies have focused on the role of oxidative stress in cholestatic hepatocellular injury and fibrosis, considering it an important contributor to the pathological process of this disease [4,5]. The mass accumulation of mitochondrial reactive oxygen species (ROS) in hepatocytes during cholestasis directly induces cell death and liver injury [6]. Furthermore, extracellular ROS derived from inflammatory cells can trigger mitochondrial dysfunction and further exacerbate oxidative stress in hepatocytes [7]. In addition, oxidative stress can also lead to lipid peroxidation, which activates hepatic stellate cells and stimulates collagen synthesis, resulting in fibrosis [8].

The major organic solutes in bile are BAs, especially hydrophobic BAs, which play important roles in the pathological process of cholestasis [9]. The infiltration and accumulation of toxic BAs in hepatocytes is the first step of cholestasis-induced liver injury. Toxic BAs not only directly kill hepatocytes through their detergent cytolytic effects causing membrane damage but also induce hepatocyte apoptosis by activating some death receptors and extrinsic/intrinsic pathways [10,11]. Furthermore, a BA-induced inflammatory response is another major contributor to cholestatic liver injury. The accumulation of BAs can increase the expression of the transcription factor through the cell surface receptor signaling pathways, sequentially stimulating the expression of downstream target genes, including a series of cytokines and adhesion molecules, with significantly enhanced neutrophil recruitment [12,13,14]. High hepatic concentrations of hydrophobic BAs can also induce mitochondria damage and activate the innate immune system, and imbalanced mitochondrial energy production further promotes the generation of ROS [15,16].

Many studies have reported that some antioxidants exert protective effects on the liver during cholestasis. For example, Vitamin E and/or C supplementation was found to significantly reduce liver fibrosis in bile duct-ligated rats [17]. *N*-acetylcysteine (NAC) not only decreased fibrosis in these rats but also prevented the increase in γ-glutamyl transpeptidase (GGT), alkaline phosphatase (ALP), and total bilirubin levels [18]. Similar to these results, after treatment with polyphenols, which are chemicals extracted from plants, a reduction in liver injury and fibrosis was also observed during cholestasis [19]. Besides these exogenous substances, the overexpression of manganese-dependent superoxide dismutase (Mn-SOD) also ameliorated liver injury and fibrosis, showing that oxidative stress-induced mitochondrial dysfunction plays an important role in cholestatic liver disease [20]. Given that extracellular ROS derived from neutrophils can trigger mitochondrial dysfunction, attenuating both intracellular and extracellular oxidative stress may more effectively inhibit cholestatic liver injury and fibrosis than attenuating either alone.

Peroxiredoxin 4 (PRDX4) is an antioxidant enzyme belonging to the PRDX family and is the only known secretory form in this family which scavenges for ROS in both intracellular and extracellular spaces [21,22]. In previous series studies, we reported that PRDX4 can protect against diabetes mellitus, atherosclerosis, insulin resistance, and nonalcoholic fatty liver disease by suppressing oxidative damage, inflammatory cytokines, and apoptotic activities, at least in part [23,24,25,26,27]. We do propose that PRDX4 locally (intracellularly) and systemically (extracellularly) plays a critical, diverse role in vivo in the protection against the initiation and development of metabolic syndrome, which is a complex, multifactorial disease, affecting not only glucose/lipid metabolism but also BA metabolism in various organs [16,21,23,24,25,26,27]. Taken together, we thus can hypothesize that PRDX4 might be able to play an important role in the pathophysiologic processes of cholestatic liver injury and to influence the progression and prognosis of cholestasis.

In the present study, we investigated the roles of PRDX4 in cholestatic liver injury by comparing the differences in pathological changes in the livers of C57BL/6J wild-type (WT) and human PRDX4 (hPRDX4) transgenic (Tg) bile duct ligation (BDL) mice. We also observed the influence of PRDX4 on the BA composition and mortality after BDL surgery.

## 2. Results

### 2.1. Reduced Liver Injury in Tg BDL Mice

**Figure 1:**
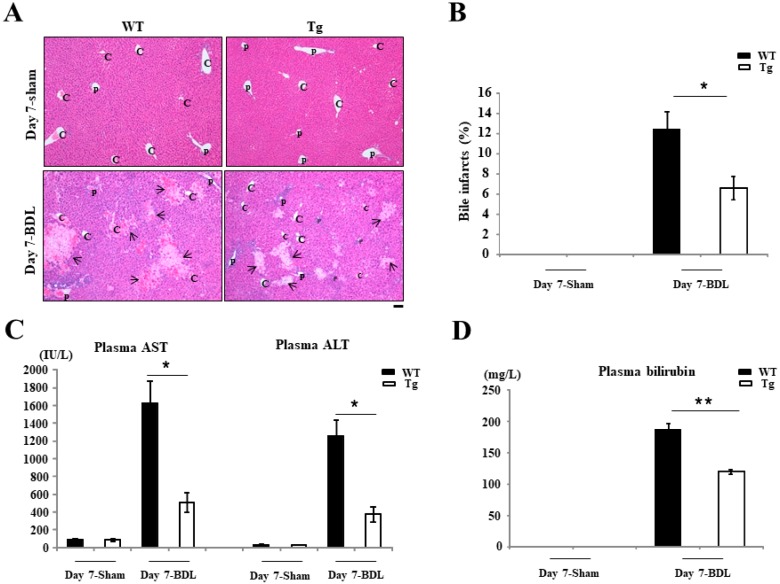
Histologic and serum analyses in mice. (**A**,**B**) The liver necrotic area of hPRDX4-Tg mice at day 7 after bile duct ligation (BDL) surgery was significantly smaller than in C57BL/6J wild-type (WT) mice. No liver necrotic areas were found in sham mice. C, central vein; P, portal vein; arrows, necrotic areas. (**C**) The serum levels of aspartate aminotransferase (AST) and alanine aminotransferase (ALT) in hPRDX4-Tg mice were significantly lower than in WT mice on day 7 after BDL surgery. (**D**) Similarly, the plasma total bilirubin level was significantly lower in PRDX4-Tg mice than in WT mice on day 7 after BDL surgery. *p* Values were calculated using Welch’s *t*-test. The values represent the mean ± SD. Bar = 100 µm * *p* < 0.05, ** *p* < 0.001, *n* = 15.

The area of bile infarct in Tg mice was significantly lower than in WT mice on day 7 after BDL surgery (12.48% ± 1.72% for WT mice versus 6.63% ± 1.17% for Tg mice; *p* < 0.05) (Figure 1A,B and Figure S1). In contrast, no control animals had any complications, and none died during the study. Consistent with the above data on the bile infarct area, the plasma levels of aspartate aminotransferase (AST) (1622 ± 249 international unit (IU)/L for the WT BDL mice versus 512 ± 109 IU/L for the Tg BDL mice; *p* < 0.05) and alanine aminotransferase (ALT) (1252 ± 186 IU/L for the WT BDL mice versus 373 ± 87 IU/L for the Tg BDL mice; *p* < 0.05) were also significantly lower in Tg mice on day 7 after BDL than in WT mice (Figure 1C), indicating less hepatocyte necrosis and cell death (apoptosis). Meanwhile, the plasma total bilirubin levels (18.8 ± 1.0 mg/dL for the WT mice versus 12.0 ± 0.4 mg/dL for the Tg mice; *p* < 0.001) in Tg mice were significantly reduced compared with WT mice on day 7 after BDL surgery (Figure 1D).

### 2.2. Attenuation of Cholestasis-Induced Oxidative Stress by the Overexpression of PRDX4

**Figure 2:**
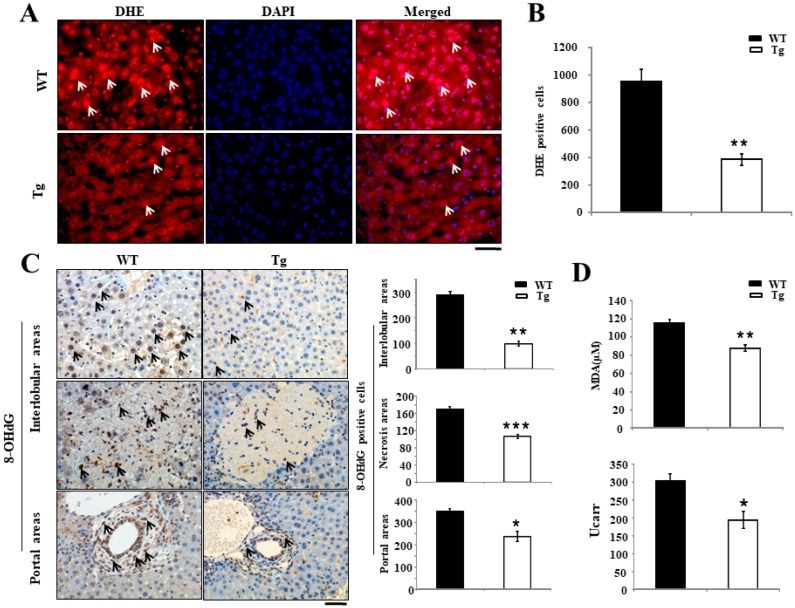
Hepatic oxidative stress in BDL-induced cholestatic liver of mice. (**A**,**B**) Dihydroethidium (DHE) staining indicates that the livers of hPRDX4-Tg mice contained significantly fewer DHE-positive hepatocytes than in WT mice at day 7 after BDL surgery. Arrows, positive cells. (**C**) Furthermore, 8-hydroxy-20-deoxyguanosine (8-OHdG) staining revealed significantly fewer numbers of accumulated 8-OHdG-positive hepatocytes in both the portal tract and interlobular areas in hPRDX4-Tg mice than in WT mice at day 7 after BDL surgery. Arrows, positive cells. (**D**) The levels of the oxidative stress markers thiobarbituric acid reactive substances (TBARS) and hydrogen peroxide (H_2_O_2_) in PRDX4-Tg mice were significantly lower than in WT mice at day 7 after BDL surgery. *p* Values were calculated using Welch’s *t*-test. The values represent the mean ± SD. Bar = 100 µm * *p* < 0.05, ** *p* < 0.001, *** *p* < 0.0001, *n* = 15.

High mRNA and protein levels of hepatic hPRDX4 and high serum levels of hPRDX4 were observed in Tg mice (Figure S2). Fluorescence microscopy revealed that there were significantly more dihydroethidium (DHE)-positive cells in Tg mice than in WT mice (955.5 ± 91.87 per 5 fields in WT mice versus 390.5 ± 41.65 per 5 fields in Tg mice; *p* < 0.001) (Figure 2A,B). Furthermore, 8-hydroxy-20-deoxyguanosine (8-OHdG) staining revealed significantly greater reductions in the numbers of accumulated 8-OHdG-positive hepatocytes in the interlobular areas, necrotic areas, and portal tract in Tg mice than in WT mice at day 7 after BDL surgery (interlobular: 289.0 ± 15.2 per 5 fields in WT mice versus 100.0 ± 8.38 per 5 fields in Tg mice; *p* < 0.001) (necrotic: 170.5 ± 5.7 per 5 fields in WT mice versus 106.6 ± 4.5 per 5 fields in Tg mice; *p* < 0.0001) (portal tract: 349.8 ± 12.8 per 5 fields in WT BDL mice versus 239.0 ± 20.8 per 5 fields in Tg BDL mice; *p* < 0.05) (Figure 2C). We also assessed the serum levels of two other oxidative stress markers: thiobarbituric acid reactive substances (TBARS) and hydrogen peroxide (H_2_O_2_). The levels of TBARS and H_2_O_2_ in Tg mice were significantly lower than in WT mice at day 7 after BDL surgery (TBARS: 115.2 ± 3.6 nM malondialdehyde (MDA) in WT BDL mice versus 87.8 ± 3.6 nM MDA in Tg BDL mice; H_2_O_2_: 302.3 ± 21.1 in WT BDL mice versus 194.7 ± 23.4 in Tg BDL mice) (Figure 2D).

### 2.3. Inhibition of Inflammation Responses in Tg BDL Mice

**Figure 3:**
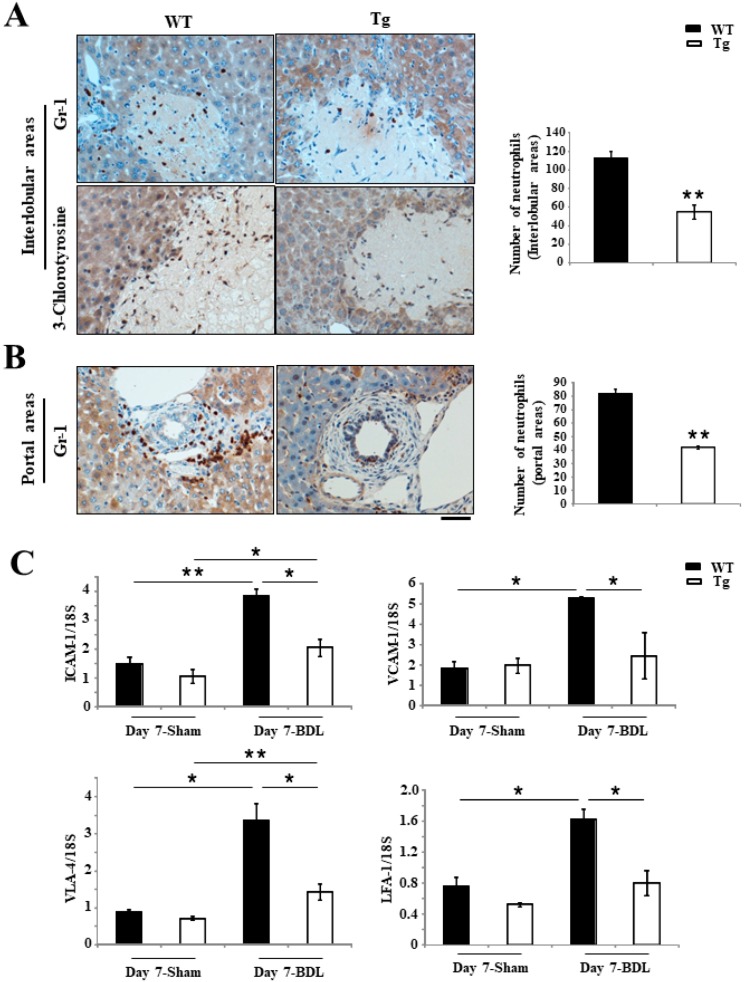
Analyses of neutrophils in BDL-induced cholestatic liver of mice. (**A**,**B**) Gr-1 staining revealed significantly fewer accumulated neutrophils in both the portal tract and interlobular areas in hPRDX4-Tg mice than in WT mice at day 7 after BDL surgery. Furthermore, chlorotyrosine-3 staining indicated that the livers of hPRDX4-Tg mice contained significantly fewer neutrophils, especially in the injured interlobular areas, than in WT mice at day 7 after BDL surgery. (**C**) Real-time reverse transcription polymerase chain reaction (RT-PCR) revealed that the expression of many adhesion molecules, such as intercellular adhesion molecule-1 (ICAM-1), vascular cell adhesion molecule-1 (VCAM-1), very late antigen-4 (VLA-4) and lymphocyte function-associated antigen-1 (LFA-1), were significantly lower in the liver of day 7 post-BDL surgery hPRDX4-Tg mice than in WT mice. This shows that the overexpression of PRDX4 suppresses the inflammatory response, thereby protecting the liver from inflammatory injury. *p* Values were calculated using Welch’s *t*-test. The values represent the mean ± SD. Bar = 100 µm * *p* < 0.05, ** *p* < 0.001, *n* = 15.

Ly-6G (Gr-1) staining showed that the accumulation of neutrophils in the injured interlobular and portal tracts of Tg mice was significantly lower than in WT mice on day 7 after BDL surgery (interlobular: 112.5 ± 7.4 per 5 fields in WT mice versus 54.7 ± 7.9 per 5 fields in Tg mice; *p* < 0.001) (Figure 3A,B); (portal tracts: 82 ± 3.2 per 5 fields in WT mice versus 42 ± 1.1 per 5 fields in Tg mice; *p* < 0.001) (Figure 3B). In addition, the staining of chlorotyrosine protein adducts revealed significantly less positive staining in Tg mice than in WT mice at day 7 after BDL (113.0 ± 6.7 per 5 fields in WT mice versus 65.5 ± 4.3 per 5 fields in Tg mice; *p* < 0.001) (Figure 3A). Real-time reverse transcription polymerase chain reaction (RT-PCR) revealed that the expression of several adhesion molecules, including intercellular adhesion molecule-1 (ICAM-1), very late antigen-4 (VLA-4), vascular cell adhesion molecule-1 (VCAM-1) and lymphocyte function-associated antigen-1 (LFA-1), were significantly down-regulated in the livers of Tg mice compared with WT mice at day 7 after BDL surgery (Figure 3C).

**Figure 4:**
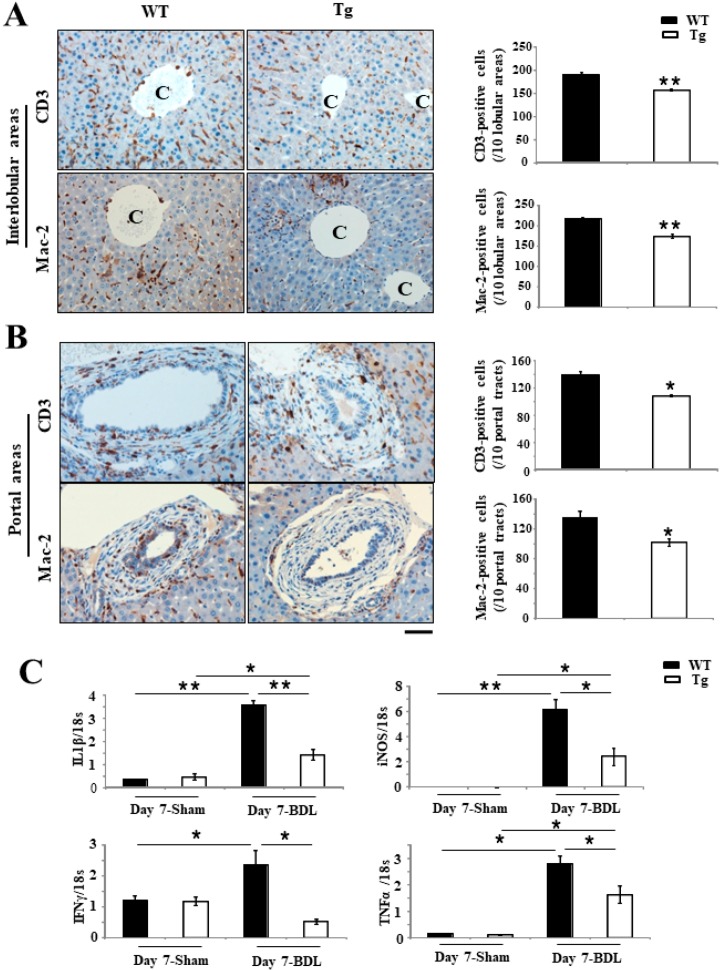
Analyses of T lymphocytes or Kupffer cells in BDL-induced cholestatic liver of mice. (**A**,**B**) Mac-2 staining revealed significantly fewer macrophages, including Kupffer cells, in both the portal tract and interlobular areas in hPRDX4-Tg mice than in WT mice at day 7 after BDL surgery. Immunohistochemistry (IHC) for CD3 also revealed significantly fewer infiltrating T lymphocytes in hPRDX4-Tg mice than in WT mice at day 7 after BDL surgery. C, central vein. (**C**) Real-time RT-PCR revealed that the expression of some proinflammatory signaling factors, such as interleukin 1 beta (IL-1β), inducible nitric oxide synthase (iNOS), interferon-γ (IFN-γ), and tumor necrosis factor-α (TNF-α), were significantly lower in the liver of day 7 after BDL surgery hPRDX4-Tg mice than in WT mice. The sham-operated group had very few inflammatory cells. *p* Values were calculated using Welch’s *t*-test. The values represent the mean ± SD. Bar = 100 µm * *p* < 0.05, ** *p* < 0.001, *n* = 15.

Furthermore, immunohistochemistry (IHC) for CD3 showed that liver injury was moderate in Tg mice, containing fewer infiltrating T lymphocytes, both per injured portal tract (139.67 ± 5.5 per 5 fields in WT mice versus 109 ± 0.58 per 5 fields in Tg mice; *p* <0.05) and interlobular area (189.67 ± 4.8 per 5 fields in WT mice versus 158 ± 1.6 per 5 fields in Tg mice; *p* <0.001) (Figure 4A,B), than in WT mice at day 7 after BDL. Similarly, staining of lectin galactoside-binding soluble 3 (Mac-2) also revealed significantly fewer macrophages, including Kupffer cells, per injured portal tract (134.2 ± 9.54 per 5 fields in WT mice versus 102.4 ± 5.04 per 5 fields in Tg mice; *p* < 0.05) and interlobular area (216.3 ± 4.6 per 5 fields in WT mice versus 173.8 ± 5.7 per 5 fields in Tg mice; *p* < 0.001) (Figure 4A,B) in Tg livers than in WT livers. Real-time RT-PCR also revealed that the expression of various proinflammatory signaling factors, such as interleukin 1β (IL-1β), inducible nitric oxide synthase (iNOS), interferon-γ (IFN-γ) and tumor necrosis factor-α (TNF-α), were significantly lower in Tg livers than in WT livers (*p* < 0.05 and *p* < 0.001, respectively) (Figure 4C).

### 2.4. Repressed Proliferation of Bile Ducts and Liver Cells in Tg BDL Mice

**Figure 5:**
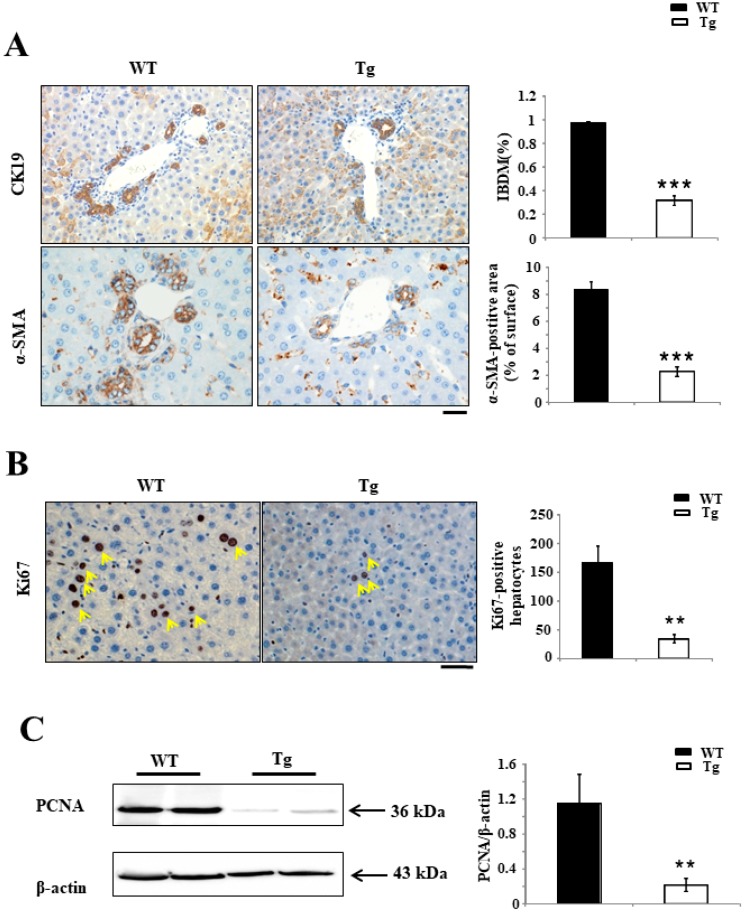
Measurements of intrahepatic bile duct mass (IBDM) and the analysis of proliferating hepatocytes in BDL-induced cholestatic liver of mice. (**A**) IBDM composed of cytokeratin 19 (CK19) bile ducts was significantly smaller in hPRDX4-Tg mice at day 7 after BDL surgery than in WT mice. Similarly, IHC of day 7 BDL hPRDX4-Tg mice livers revealed a significantly lower expression of α-smooth muscle actin (α-SMA) along the hyperplastic bile than in WT mice. (**B**) The number of Ki67^+^ hepatocytes was significantly lower in hPRDX4-Tg mice at day 7 after BDL surgery than in WT mice. Arrows, positive cells. (**C**) Corresponding to the histologic data, a Western blotting analysis revealed that the expression of proliferating cell nuclear antigen (PCNA) in livers of hPRDX4-Tg mice at day 7 after BDL surgery was significantly lower than in WT mice. Because the aberrant regeneration (proliferation) of hepatocytes is closely related to the extent of liver injury, we can conclude that the overexpression of PRDX4 suppressed the proliferative activity of hepatocytes and thereby protected the liver from injury. *p* Values were calculated using Welch’s *t*-test. The values represent the mean ± SD. Bar = 100 µm. ** *p* < 0.001, *** *p* < 0.0001, *n* = 15.

The results of cytokeratin 19 (CK19)^+^ staining indicate that the intrahepatic bile duct mass (IBDM), composed of CK19^+^ large–to–small bile ducts, was significantly smaller in Tg mice than in WT mice at day 7 after BDL surgery (0.96% ± 0.01% in WT mice versus 0.32% ± 0.04% in Tg mice; *p* < 0.0001). In addition, IHC staining of the hyperplastic bile ductules and the hepatic sinusoids in the portal and periportal tracts revealed significantly fewer α-smooth muscle actin (SMA)-positive SMCs in Tg livers than in WT livers (8.32% ± 0.63% in WT mice versus 2.29% ± 0.37% in Tg mice; *p* < 0.0001) (Figure 5A). Ki67 staining was used to detect proliferative activity, and the results indicated that the number of Ki67^+^ liver cells was significantly fewer in Tg mice at day 7 after BDL surgery than in WT mice (167 ± 29.4 per 5 fields in WT mice versus 35 ± 7 per 5 fields in Tg mice; *p* < 0.001) (Figure 5B). Similarly, Western blotting showed that the expression of proliferating cell nuclear antigen (PCNA) was significantly downregulated in the livers of Tg mice compared with WT mice on day 7 after BDL surgery (Figure 5C).

### 2.5. An Improved Prognosis of BDL-Induced Cholestasis in Tg Mice Compared with WT Mice

**Figure 6:**
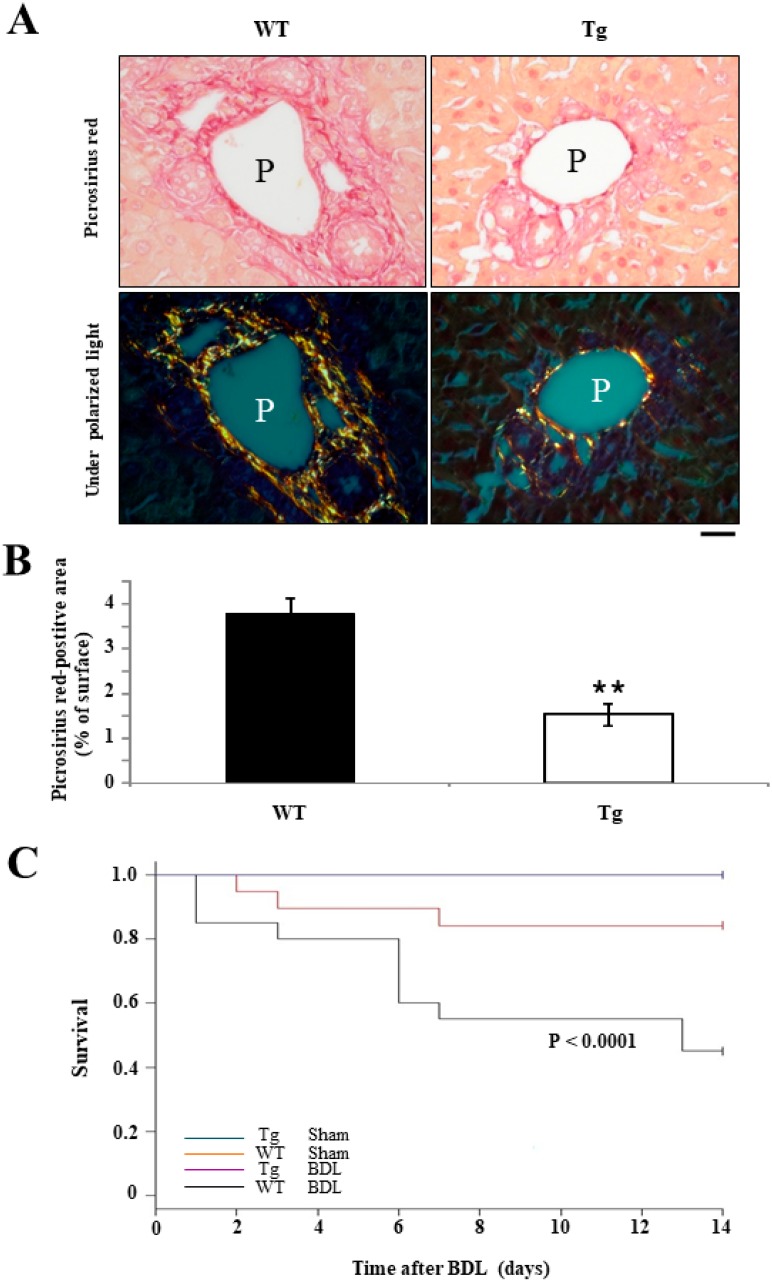
Analyses of peribiliary fibrosis and mortality in BDL mice. (**A**) Picrosirius red staining revealed evidence of significantly greater peribiliary fibrosis in WT mice on day 7 after BDL surgery than in hPRDX4-Tg mice. P, portal vein. (**B**) The mortality rate was significantly lower in hPRDX4-Tg mice on day 7 after BDL surgery than in WT mice; nevertheless, there were no deaths in the control group during the study period. *p* Values were calculated using Welch’s *t*-test. The values represent the mean ± SD. Bar = 100 µm ** *p* < 0.001, *n* = 15.

Picrosirius red staining results suggest that the degree of fibrosis around the bile ducts of Tg mice was significantly lower than in those of WT mice at day 7 after BDL surgery (8.32% ± 0.63% in WT mice versus 2.29% ± 0.37% in Tg mice; *p* < 0.001) (Figure 6A,B), which echoes the staining results of IBDM and α-SMA IHC. The survival rate of Tg mice was significantly higher than that of WT mice on day 7 after BDL surgery (Figure 6C).

### 2.6. Changes in the BA Metabolism in Tg Mice

**Figure 7:**
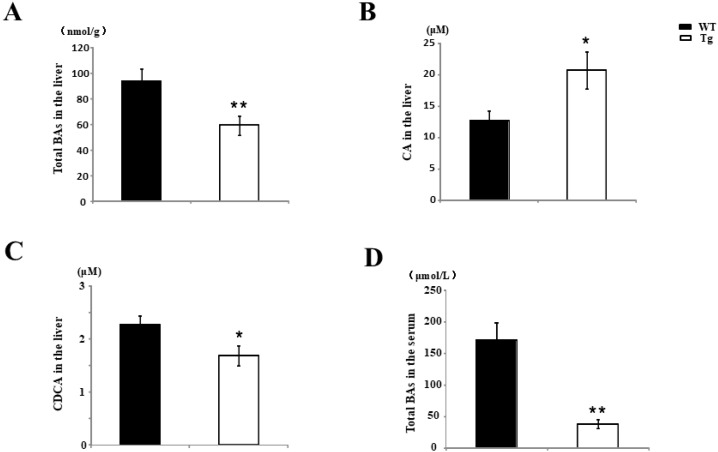
Changes of BA metabolism in Tg mice. The total BA hepatic (**A**) and plasma (**D**) levels in hPRDX4-Tg mice were significantly lower than in WT mice on day 7 after BDL surgery. The hepatic cholic acid (CA) level was lower while the hepatic chenodeoxycholic acid (CDCA) level was higher in Tg BDL mice than in WT mice. *p* Values were calculated using Welch’s *t*-test. The values represent the mean ± SD. * *p* < 0.05, ** *p* < 0.001, *n* = 15.

Hepatic levels of BAs (93.76 ± 9.6 nM/g for the WT mice versus 59.4 ± 7.4 nM/g for the Tg mice) (Figure 7A) in Tg mice were significantly lower than in WT mice on day 7 after BDL surgery. Interestingly, higher hepatic cholic acid (CA) levels (Figure 7B) and lower hepatic chenodeoxycholic acid (CDCA) levels (Figure 7C) were observed in Tg BDL mice than in WT BDL mice. Furthermore, the plasma BA concentration (171.83 ± 28.28 μM/L for the WT mice versus 38.11 ± 7.47 μM/L for the Tg mice) was lower in Tg BDL mice than in WT BDL mice (Figure 7D). These findings indicate that PRDX4-Tg can improve the overall condition of cholestasis in mice.

## 3. Discussion

**Figure 8:**
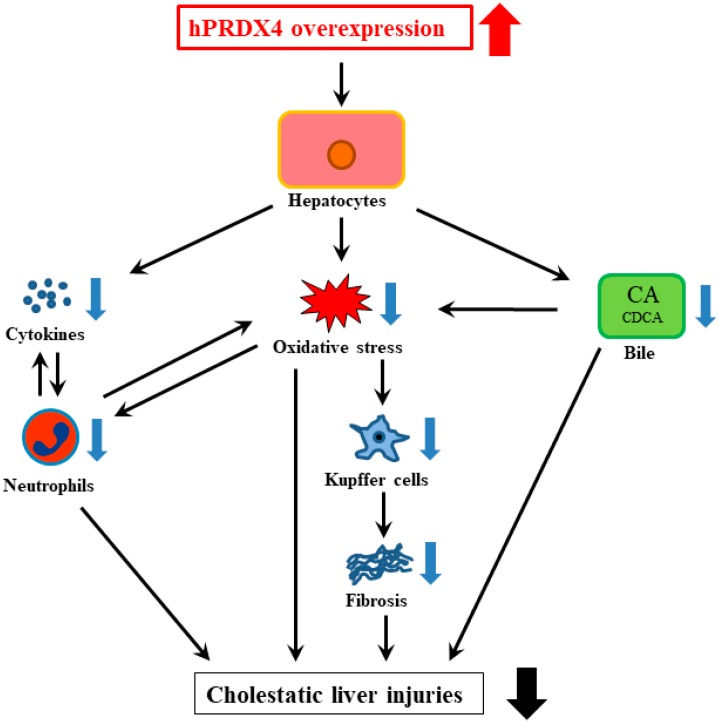
A schematic illustration of the critical roles of PRDX4 in BDL-induced cholestasis.

In this study using a mouse model of BDL-induced cholestatic liver injury, we demonstrated that the overexpression of hPRDX4 significantly reduced cholestasis-related liver damage and fibrosis and improved the total survival of mice after BDL surgery, suggesting that PRDX4 may play a crucial role in the pathological processes of obstructive cholestatic hepatitis (Figure 8).

A number of different pathologies may cause a reduction in the bile flow and subsequent induction of cholestasis in humans, including defective bile export, gallstones, and liver tumor [28]. However, regardless of the triggers, cholestasis often results in a variety of similar pathological changes, including hepatocellular injury and necrosis, inflammatory cell infiltration, proliferation of bile ducts, and fibrosis [29,30,31]. Surgical BDL in mice is a common experimental model for inducing obstructive cholestatic liver injury and leads to a stereotypical histopathological phenotype very similar to that of human cholestasis. In the present study, seven days after surgical BDL, the impaired bile flow caused a significant increase in the AST and ALT serum levels, large areas of liver tissue necrosis, massive neutrophil infiltration with necroinflammation, cholangiocyte and liver cells proliferation, the activation of macrophages and Kupffer cells, and enhanced fibrosis in mice.

Oxidative stress has been confirmed to occur in animal models of cholestatic liver injury and plays important roles in the pathological process during cholestasis [32]. In the present study, as expected, these pathological changes were significantly reduced in Tg mice, showing that a high PRDX4 expression can suppress the progression of this disease. The overexpression of PRDX4 reduced the number of 8-OHdG-positive cells in the liver, thereby protecting liver cells from oxidative stress-induced injury. Furthermore, high levels of PRDX4 were also found in the serum of mice as a secretory antioxidant, reducing the serum levels of TBARS and H_2_O_2_; this suggests that PRDX4 also significantly decreases oxidative stress in extracellular spaces, as shown in our previous series of studies [23,24,25,26,27]. PRDX4 protecting hepatocytes seems to lead directly to a reduction in the necrosis area and subsequent proliferation and fibrosis in the liver of TG BDL mice. However, an increase in cholestasis-induced intracellular oxidative stress is rarely sufficient to cause cell injury directly [33]. A study reported that the expression of mitochondrial Mn-SOD reduced liver injury and fibrosis, but the expression of cytosolic Cu/Zn-SOD was largely ineffective, showing that mitochondrial dysfunction and depolarization is a main cause of cell injury [20]. Neutrophil-generated ROS, including H_2_O_2_ (the main metabolic substrate for PRDX4), generate extracellular oxidant stress and diffuse into a target cell to trigger mitochondrial dysfunction and oncotic necrosis [34]. Therefore, in the present study, PRDX4 may have attenuated cholestasis-induced liver injury by reducing extracellular oxidant stress and mitochondrial dysfunction, thereby suppressing the release of inflammatory cytokines and neutrophil infiltration. Extracellular PRDX4 may play a larger role in protecting against cholestasis-induced liver injury. Taken together, these findings suggest that PRDX4 may be more effective as a cholestasis therapy than other antioxidants.

Generally, surgical BDL is associated with a relatively high mortality rate because of infectious complications, such as bile leakage and rupture of the gallbladder [35]. In the present study, a mortality rate of approximately 40% in WT mice was observed in the first week after surgery, which is different from other studies showing lower mortality rates, probably due to differences in the mouse strain used, surgical conditions, or technical inaccuracies [36]. However, despite these objective factors being the same for all mice in our study, a significant difference was noted in the mortality rate between WT and Tg mice, with Tg mice showing a significant lower mortality than WT mice at one week after BDL surgery. The relatively mild liver injury in Tg mice may explain this phenotype. However, liver injury is rarely a direct reason for death in mice a short period after surgical BDL. It was therefore very difficult to confirm the exact mechanism underlying the lower mortality in Tg mice, especially since severe liver injury was found in all BDL mice compared to sham mice. Notably, fewer foci of hepatic damage with a reduction in the number of immune activated cells were also observed in the interlobular and portal areas of BDL Tg mice. The infiltration of immune activated cells is deemed to be associated with bacterial growth in bile and liver extracts that triggers a local and systemic response and induces septicemia, septic shock, and multiple-organ failure, finally resulting in death; this resembles severe acute cholangitis with a high mortality in both humans and mice [37,38]. The reduced expression of pro-inflammatory cytokines, such as IL-1β, IFN-γ and TNF-α, may also reflect the fact that bacterial infection was suppressed in BDL Tg mice [12,39]. A reduced bacterial growth may therefore be the main reason for the lower mortality in Tg mice than in WT mice in the present study. Our results may have potential clinical implications for increasing the survival of patients with cholestasis.

BAs, which are a major component in bile and are synthesized from cholesterol in the liver, are a crucial factor during cholestasis-induced liver injury. Under cholestasis conditions, the exposure of hepatocytes to high concentrations of BAs causes a series of pathophysiological changes, including mitochondrial dysfunction, inflammatory response, and oxidative stress, thereby leading to liver injury and fibrosis [40]. In the present study, we observed a significant decrease in total hepatic and serum concentrations of BAs in BDL Tg mice at seven days after surgery compared to WT mice. Tg BDL mice showed milder liver injury than WT BDL mice, probably because of the reduction in BA accumulation. However, details regarding the mechanism underlying this phenomenon remain unclear, as BA synthesis is tightly regulated by a complex but integrated network of mechanisms that are not completely understood. The enterohepatic circulation of BAs can control BA synthesis in the liver by a negative feedback mechanism and maintain a constant BA level [41]. We previously reported that the overexpression of PRDX4 can affect the function of intestinal epithelium by changing the expression of lipid and BA metabolism-related genes [42]. The improved intestine-to-liver signaling pathway in Tg mice may help reduce the size of the BA pool, thereby reducing BA synthesis in the liver through feedback regulation, which helps lower the level of total BAs in the liver and serum of Tg mice.

A large amount of BAs accumulate in the liver during cholestasis, but not all BAs have a cytotoxic effect on hepatocytes; such an effect is determined by the physical properties and physiological function of these amphipathic molecules, which are closely associated with their chemical structure [43]. The most important of these factors is the hydrophobicity of BAs [9]. Secondary BAs cannot be generated in the intestine via bacterial biotransformation after BDL, so we examined the concentration of two primary BAs: CA and CDCA. CDCA is a hydrophobic BA which causes liver damage, whereas CA is a hydrophilic BA which reduces anti-inflammatory and anti-oxidative stress on hepatocytes during cholestasis [44]. Surprisingly, we found that the levels of the hydrophobic CDCA were lower while those of the hydrophilic CA were higher in the liver of Tg BDL mice than in WT BDL mice. Given our previous series results, we speculate that the high expression of PRDX4 affects the lipid and BA metabolism in the body as a whole, likely via enterohepatic circulation.

Several limitations associated with the present study warrant a mention. Firstly, although we feel that the total number of experimental mice (*n* = 100–120) was sufficient to explain the phenotype of Tg mice, only one model that mimics cholestatic liver injury (BDL) was used. BDL leads to an acute biliary obstruction, which is usually used to observe necroinflammation, cholangiocyte proliferation, and portal fibrosis. More observations of PRXD4 in other rodent cholestasis models, such as 3,5-diethoxycarbonyl-1,4-dihydrocollidine (DDC) or α-Naphthyl-isothiocyanate (ANIT)-induced cholestatic liver injury, where the early and chronic pathological alterations can be evaluated [45,46], will help identify the specific roles of PRXD4 at different stages and its effects on pathological changes during cholestasis. Secondly, given that observations in BDL mice were made at only one point in time (day 7), the roles of PRDX4 in secondary biliary chronic liver disease, which occurs from 14 days after BDL surgery, were ignored. Thirdly, since the present study mainly focused on oxidative stress, necroinflammation, bile ducts proliferation, and fibrosis, although a significant difference was found in hepatic content and the composition of BAs between WT and Tg mice, a number of important proteins related to the synthesis and transport of BAs were not examined. This will be addressed in future studies.

In conclusion, in this study, hPRDX4 Tg mice showed a beneficial phenotype that reduced intracellular and extracellular oxidative stress, suppressed inflammatory cells infiltration, and improved the BA metabolism system, leading to smaller necrosis hepatic areas and attenuated liver injury, with a lower mortality after surgery in BDL-induced cholestatic liver injury. All of these features suggest that activators of PRDX4 may be a novel treatment for ameliorating the severity of cholestatic liver injury and improving the prognosis of patients with cholestasis.

## 4. Materials and Methods

### 4.1. Animals and BDL Model

Tg mice were generated on a C57BL/6 background [20,21,22,23]. Male WT (8 weeks old, 22–25 g) and Tg (8 weeks old, 22–25 g) were used for the experiments. Mice were housed in a standard facility that was temperature-controlled with a 12 h light/dark cycle, standard rodent chow, and water ad libitum. In order to obtain a cholestatic liver injury model, we performed BDL in mice. After the mice were anesthetized (intraperitoneal injection of ketamine (100 mg/kg) (Daiichi Sankyo Co., Tokyo, Japan) and medetamidine (2 mg/kg; Meiji Yakuhin Co., Tokyo, Japan), the abdomen was opened, and the cutis plus fascia were cut at the same time with surgical scissors. The common bile duct was double-ligated with sterile surgical 7-0 silk sutures and cut between the ligatures. Both abdominal layers were closed with 6-0 Mersilk. As controls, the sham-operated animals also had their abdomen opened to expose the bile duct without ligation. At seven days after BDL or sham surgery, mice were anesthetized by an injection of ketamine-medetamidine and euthanized by exsanguination. In all animals, blood samples were taken from the infraorbital vein, frozen, and used for various experiments. After the blood was removed, the liver was excised and cut into small pieces, frozen, and fixated in 10% neutral-buffered formalin for the various experiments described below.

The Ethics Committee of Animal Care and Experimentation, Kanazawa Medical University, Japan, approved the protocols. The project code of the approval, 2017-128, was identified on 20 December 2017. They were performed according to the Institutional Guidelines for Animal Experiments and the Law (no. 105) and Notification (no. 6) of the Japanese government.

### 4.2. Histopathology

After fixation in 10% neutral-buffered formalin for 24 h, both BDL-induced cholestatic liver specimens and sham-operated liver specimens were stained with hematoxylin and eosin (H&E), picrosirius red stain or IHC preparations in sequential sections.

Paraffin-embedded liver specimens were systematically cut into sequential 4-μm-thick sections. For a quantitative analysis, images of H&E-stained and special stained sections or IHC sections were captured and evaluated by the NanoZoomer Digital Pathology Virtual Slide Viewer software program, version 2.0 (Hamamatsu Photonics Corp., Hamamatsu, Japan). The areas of bile infarcts (hepatic necrosis) were measured by H&E-stained sections and calculated as the percentage of the necrotic area divided by the total area. Picrosirius red staining (Picro-Sirius Red Stain Kit; ScyTek Laboratories, Inc., Logan, UT, USA) was used to quantify the liver fibrosis in 5 randomly selected fields of portal and periportal tracts per section (original magnification, 400×). Under yellow birefringence polarized light, fibers around periportal tracts stained red were defined as expressing collagen type I. For single images, the proportions of collagen content were measured, and large bile ducts (>15 mm in diameter) and vessels were excluded.

### 4.3. Analyses of Liver Injury Induced by Cholestasis

Serum AST, ALT, and total bilirubin levels were determined using commercial assay kits (Wako Pure Chemical Co., Osaka, Japan). Total BA levels in the liver were also determined using commercial assay kits (Wako Pure Chemical Co.). CA and CDCA levels were determined using CA or CDCA ELISA kits, respectively (Cell Biolabs, Inc., San Diego, CA, USA).

### 4.4. Histology and IHC

For histopathological analyses, 15 mice were selected from each group, and one representative section per mouse was prepared for IHC staining. To analyze the ROS/oxidative stress levels in BDL-induced cholestasis tissues, the liver sections were stained with an anti-8-OHdG monoclonal antibody (1:200; Japan Institute for the Control of Aging, Fukuroi, Japan). To observe the small and large bile duct liner, we used a goat polyclonal anti-mouse CK19 antibody (1:50; Vector Laboratories, Inc., Burlingame, CA, USA). In several subsequent analyses, we differentiated bile ducts by size (≤15 μm in diameter, small; >15 μm in diameter, large). Around the portal tracts, hyperplastic and proliferating bile ductules were able to be distinguished. The IBDM was calculated as follows: area occupied by CK19^+^ bile ducts/total area × 100.

To evaluate the intensity of inflammation, we used a polyclonal rabbit anti-human CD3 antibody (1:1; Dako Cytomation Co., Tokyo, Japan), a rat anti-mouse Gr-1 antibody (Ly-6G; 1:100; Birmingham, AL, USA) and a rat anti-mouse Mac-2 monoclonal antibody (1:1000; Vector Laboratories, Inc.). In addition, we used a rabbit antichlorotyrosine polyclonal antibody (1:25; Hycult Biotech, Uden, The Netherlands) to distinguish the protein adducts of chlorotyrosine. For each section, we randomly selected five fields of portal and interlobular areas and counted the numbers of positive T lymphocytes, neutrophils, and macrophages (Kupffer cells) (original magnification, 400×).

To evaluate the severity of peribiliary fibrogenic of BDL-induced portal tracts on day 7, a monoclonal mouse antihuman a-SMA antibody (1:1000; Dako) was used. In addition, Ki67 (MIB-1; 1:2000; Epitomics, Cambridge, UK) rabbit monoclonal antibody was used to analyze the bile duct proliferative activity.

### 4.5. ROS Detection

The oxidation-sensitive fluorescent dye DHE (Molecular Probes, Eugene, OR, USA) was used to assess the intracellular ROS levels at seven days after bile duct ligation. We counted the number of positive cells in 5 randomly selected fields per section (original magnification, 400×). In order to assess lipid peroxidation, we use the well-established TBARS method, which can also be used to determine the oxidative stress. The TBARS Assay Kit (Cayman Chemical Company, Ann Arbor, MI, USA) was used to measure the TBARS levels in serum collected from BDL-induced cholestasis mice. Results were expressed as nanomoles of MDA. For the assessment of the hydroperoxide levels, a diacron reactive oxygen metabolites (dROM) test was performed using a FRAS4 System (H&D, Parma, Italy), as described previously [26]. This measurement was expressed in Ucarr (Carratelli unit) values, with 1 unit corresponding to 0.8 mg/L H_2_O_2_.

### 4.6. RT-PCR and Real-Time RT-PCR

RT-PCR and real-time RT-PCR were used to analyze the gene expression in the liver on day 7 after BDL surgery. Total RNA was extracted from mouse liver using the Total RNA Extraction Miniprep System (Viogene BioTek, New Taipei City, Taiwan). The whole extraction process was performed under RNase-free conditions in order to prevent RNA degradation. Custom made primers and TaqMan probe for employed genes amplification were purchased from Life Technologies. The relative expression of each gene was normalized to that of 18S ribosomal RNA using random primers.

### 4.7. Western Blotting

Proteins (20 mg) isolated from individual mouse liver tissue samples were separated by SDS-PAGE and transblotted onto Immun-Blot polyvinylidene difluoride membranes (Bio-Rad Laboratories, K.K., Tokyo, Japan). The membranes were then incubated overnight at 4 °C with a PCNA (1:200; Santa Cruz Biotechnology, Dallas, TX, USA) and a mouse anti-chicken monoclonal β-actin antibody (1:1000; Santa Cruz Biotechnology) diluted in Can Get Signal solution 1 (Toyobo, Osaka, Japan), after which the membranes were incubated for 1 h at room temperature with a horseradish peroxidase conjugated goat anti-rabbit antibody (Vector Laboratories).

### 4.8. Statistical Analyses

Results were expressed as the means ± standard deviation. Significant differences were analyzed using Student’s or Welch’s *t*-test or an analysis of variance where appropriate. Values of *p* < 0.05 were considered statistically significant.

## Supplementary Materials

Supplementary materials can be found at http://www.mdpi.com/1422-0067/19/9/2509/s1.

## Abbreviations

PRDX4	Peroxiredoxin 4
ER	Endoplasmic reticulum
WT	Wild-type
UDCA	Ursodeoxycholic acid
BDL	Bile duct ligation
ROS	Reactive oxygen species
BAs	Bile acids
NAC	N-acetylcysteine
GGT	γ-Glutamyl transpeptidase
ALP	Alkaline phosphatase
Mn-SOD	Manganese-dependent superoxide dismutase
DHE	Dihydroethidium
TBARS	Thiobarbituric acid reactive substances
MDA	Malondialdehyde
ALT	Alanine aminotransferase
AST	Aspartate aminotransferase
IL-1β	Interleukin 1 β
iNOS	Inducible nitric oxide synthase
IFNγ	Tumor necrosis factor-α
TNF-α	Tumor necrosis factor-α
CA	Cholic acid
CDCA	Chenodeoxycholic acid
8-OHdG	8-hydroxy-20-deoxyguanosine
CK19	Cytokeratin 19
IBDM	Intrahepatic bile duct mass
α-SMA	α-Smooth muscle actin
LDL	Low-density lipoprotein
dROM	Diacron reactive oxygen metabolites
PCNA	Proliferating cell nuclear antigen
DDC	3,5-Diethoxycarbonyl-1,4-dihydrocollidine
ANIT	α-Naphthyl-isothiocyanate
