# Supplementary material for: Protective Effects of Peroxiredoxin 4 (PRDX4) on Cholestatic Liver Injury
Source: Int J Mol Sci. 2018 Aug 24;19(9):2509. doi: 10.3390/ijms19092509 (PMC6163182; doi:10.3390/ijms19092509)
Supplement: Supplementary file 1 [file ijms-19-02509-s001.zip › ijms-344736-SI.pdf]

Supplementary materials

Protective Effects of Peroxiredoxin 4 (PRDX4) on Cholestatic Liver Injury

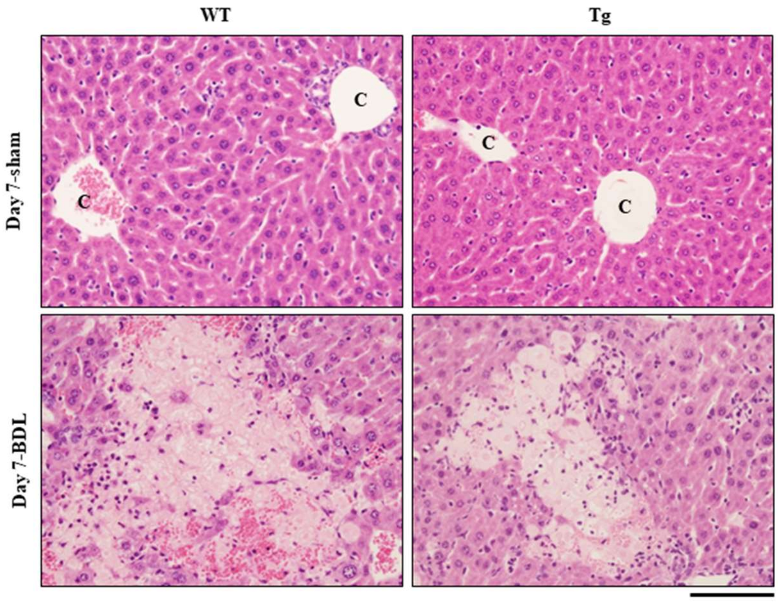

Figure S1. Histologic analyses in the liver of mice. Representative photomicrographs (high-power view) of H&E stained sections in the liver of WT or Tg mice on day 7 after sham or BDL surgery. Bar = 100 μm, *n* = 15. C, central vein.

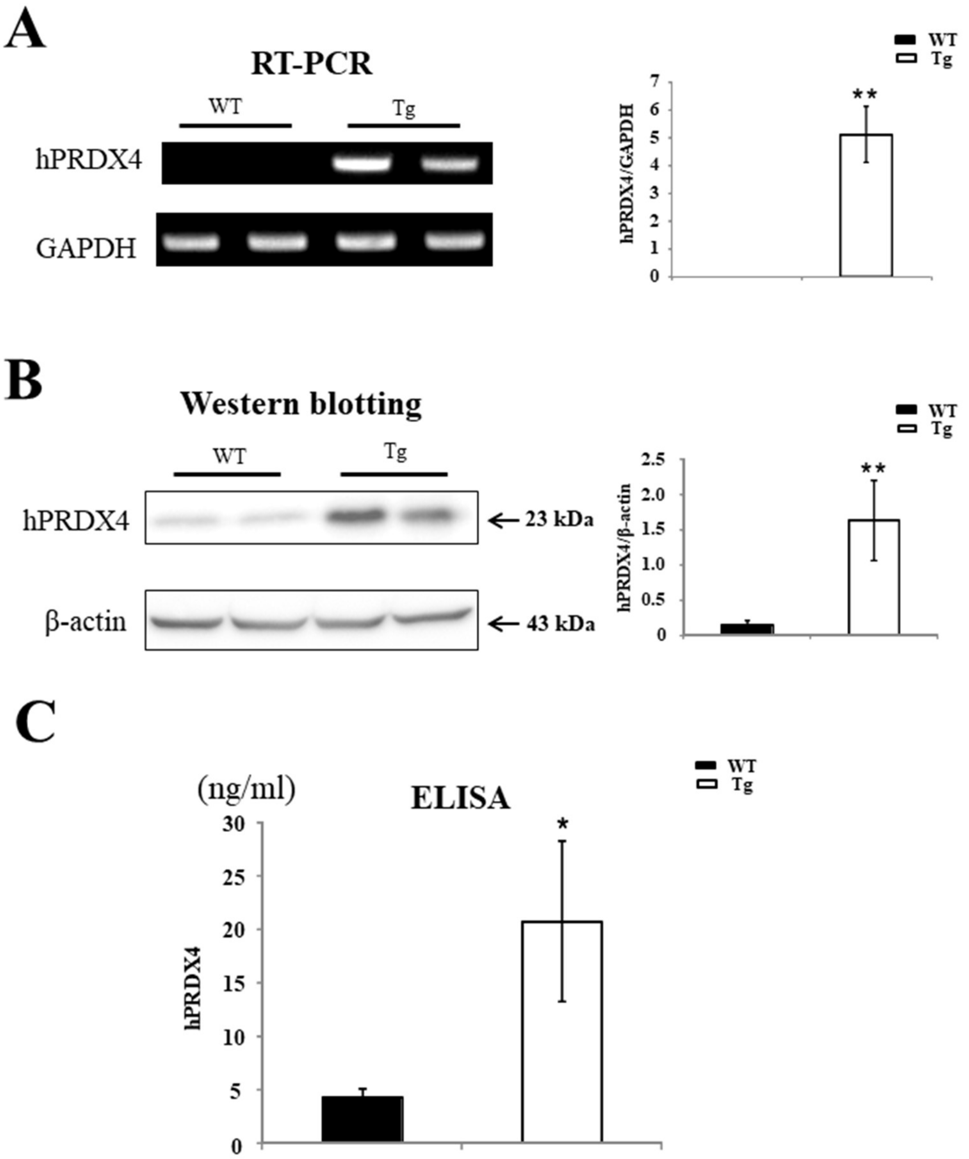

Figure S2. The expression of hPRDX4 in BDL mice. The mRNA and protein expression of hPRDX4 in the liver of WT or *hPRDX4*$^{+/+}$ mice were examined by RT-PCR (**A**) and Western blotting (**B**), respectively. (**C**) The serum hPRDX4 level was measured by an ELISA kit. p values were calculated using Welch's t-test. The values represent the mean ± SD. * $p < 0.05$, ** $p < 0.001$, $n = 5$.
